# Supplementary material for: Estimating total human deaths from rabies: A statistical framework leveraging reports of rabies transmission from deceased organ and tissue donors
Source: PLoS Negl Trop Dis. 2026 Sep 21;20(9):e0014731. doi: 10.1371/journal.pntd.0014731 (PMC13592719; doi:10.1371/journal.pntd.0014731)

Switzerland (canine−rabies−free)

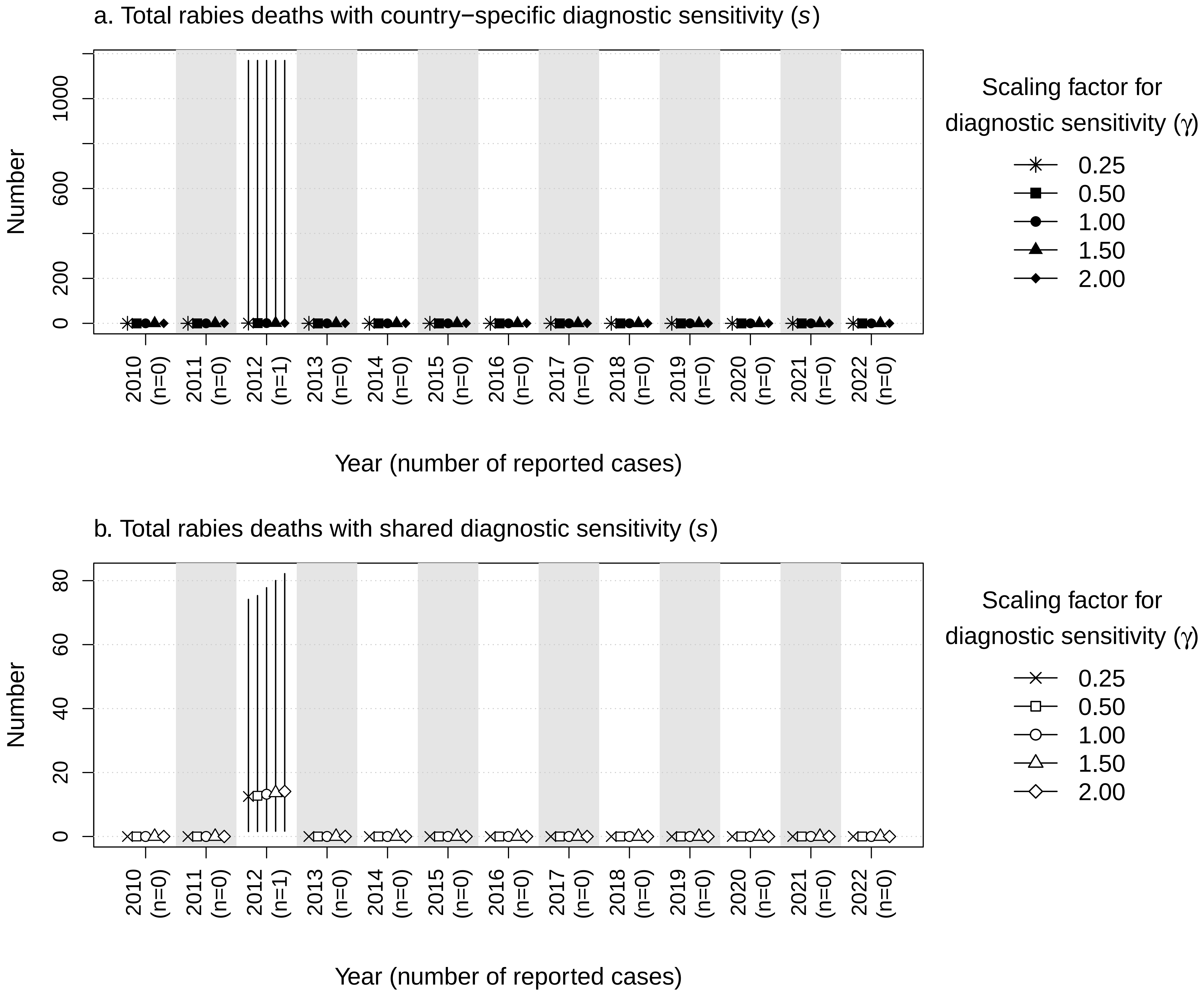

Supplement: Supplementary file 6 [file pntd.0014731.s005.zip › Result_B/Switzerland/Figure_S3_Switzerland.pdf]
